# Causal effect of gut microbiota on pancreatic cancer: A Mendelian randomization and colocalization study

**DOI:** 10.1111/jcmm.18255

**Published:** 2024-03-25

**Authors:** Xin Li, Zhihai Liang

**Affiliations:** ^1^ Department of Gastroenterology, The First Affiliated Hospital Guangxi Medical University Nanning China

**Keywords:** colocalization, gut microbiota, Mendelian randomization, pancreatic cancer, summary data‐base Mendelian randomization

## Abstract

The causal relationship between gut microbiota (GM) and pancreatic cancer (PC) remains unclear. This study aimed to investigate the potential genes underlying this mechanism. GM Genome‐wide association study (GWAS) summary data were from the MiBioGen consortium. PC GWAS data were from the National Human Genome Research Institute‐European Bioinformatics Institute (NHGRI‐EBI) GWAS Catalogue. To detect the causal relationship between GM and PC, we implemented three complementary Mendelian randomization (MR) methods: Inverse Variance Weighting (IVW), MR‐Egger and Weighted Median, followed by sensitivity analyses. Furthermore, we integrated GM GWAS data with blood cis‐expression quantitative trait loci (eQTLs) and blood cis‐DNA methylation QTL (mQTLs) using Summary data‐based Mendelian Randomization (SMR) methods. This integration aimed to prioritize potential GM‐affecting genes through SMR analysis of two molecular traits. PC cis‐eQTLs and cis‐mQTLs were summarized from The Cancer Genome Atlas (TCGA) data. Through colocalization analysis of GM cis‐QTLs and PC cis‐QTLs data, we identified common genes that influence both GM and PC. Our study found a causal association between GM and PC, including four protective and five risk‐associated GM [Inverse Variance Weighted (IVW), *p* < 0.05]. No significant heterogeneity of instrumental variables (IVs) or horizontal pleiotropy was found. The gene SVBP was identified as a GM‐affecting gene using SMR analysis of two molecular traits (FDR<0.05, P_HEIDI>0.05). Additionally, two genes, MCM6 and RPS26, were implicated in the interaction between GM and PC based on colocalization analysis (PPH4>0.5). In summary, this study provides evidence for future research aimed at developing suitable therapeutic interventions and disease prevention.

## 1 BACKGROUND

Pancreatic cancer (PC) has a poor prognosis with few effective therapeutic options.^1^ The incidence of PC is persistently rising, with little improvement in survival rates.^2^ For pancreatic ductal adenocarcinoma (PDAC), the overall 5‐year relative survival rate at diagnosis was 8.5%.^3^ However, early detection and prompt intervention can significantly extend the survival time of patients, offering a greater chance for long‐term survival.^4^ As a result, it is urgent to elucidate the underlying aetiology of PC to make progress in its diagnosis, prevention and treatment.

The pancreas is anatomically connected to the gastrointestinal tract via the pancreatic duct.^5^ This connection may facilitate the potential reflux of microbiota into the pancreatic duct, a phenomenon that is corroborated by the higher incidence of PDAC in the head of the pancreas, as opposed to its body or tail.^6^ A disrupted gut microbiota (GM) can lead to chronic inflammation, which plays a role in the pathogenesis of PC.^7^

Mendelian randomization (MR) is a method using genetic variants associated with a risk factor as an exposure to assess whether there is a causal effect on outcomes, minimizing the impact of external confounders.^8^, ^9^ Moreover, these genetic variants can potentially regulate DNA methylation (DNAm), gene expression, protein levels and the abundance of GM.^10^, ^11^ Currently, MR methods have been increasingly applied to investigate the causal relationship between GM and PC.^12^, ^13^, ^14^, ^15^, ^16^ However, the genes potentially involved in interactions between GM and PC remain poorly understood. To address this, Summary data‐based Mendelian Randomization (SMR) methods have been developed. SMR that integrates Genome‐wide association study (GWAS) data with eQTLs has been developed to prioritize causal variants mediated by gene expression.^11^ To date, there have been few MR studies that integrated GM GWAS and blood QTLs reported.^17^

In our study, we explored the causal relationship between GM and PC using the MR methods. Then we integrated the GM GWAS summary data with eQTLs and mQTLs in the blood tissue by SMR methods. Furthermore, we uncovered the potential common genes between GM and PC through colocalization analysis, improving our genetic insight into their relationship.

## 2 MATERIALS AND METHODS

### 2.1 Study design

**FIGURE 1:**
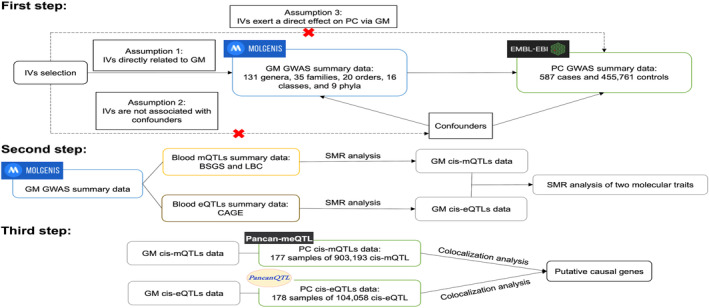
Study flowchart.

This study aimed to establish a causal link between GM and PC and to explore potential genes involved in this mechanism. Firstly, we utilized MR analysis to assess the causal impact of GM on PC. Secondly, SMR analysis was employed to identify potential GM cis‐eQTLs and GM cis‐mQTLs. Then we investigated the pleiotropic associations via GM cis‐mQTLs and GM cis‐eQTLs to pinpoint genes that were impacted by GM across two molecular traits. Thirdly, we conducted the colocalization analysis to identify shared genes between GM and PC. The study flowchart is shown in Figure 1.

### 2.2 Data sources

In this study, all data were obtained from public databases; therefore, neither additional ethical approval nor participants' informed consent was required.

The summary data for GM GWAS were from a meta‐analysis by the MiBioGen consortium(https://mibiogen.gcc.rug.nl/), which encompassed 18,340 individuals predominantly of European descent (*n* = 13,266) across 24 cohorts, and included a total of 211 taxa, spanning 131 genera, 35 families, 20 orders, 16 classes, and nine phyla. The study coordinated 16S ribosomal RNA gene sequencing with genotyping data for all participants, contributing to the identification of host factors influencing human GM composition.

The summary data for PC GWAS, obtained from the NHGRI‐EBI GWAS Catalogue, included 587 European ancestry cases and 455,761 European ancestry controls.^18^

The PC cis‐eQTLs data were from http://gonglab.hzau.edu.cn/PancanQTL/ with 178 samples of 104,058 cis‐eQTLs from The Cancer Genome Atlas (TCGA) data portal (https://tcga‐data.nci.nih.gov/tcga/).^19^

The PC cis‐mQTLs data were from http://gonglab.hzau.edu.cn/Pancan‐meQTL/ with 177 samples of 903,193 cis‐mQTLs from TCGA data portal (https://tcga‐data.nci.nih.gov/tcga/).^20^ DNA methylation quantitative trait loci (mQTLs) are regions of the genome containing DNA sequence variants that influence the methylation levels [Single nucleotide polymorphism (SNPs) affect local methylation levels of CpG sites].

The peripheral blood eQTLs data were from the Consortium for the Architecture of Gene Expression eQTLs summary data^21^ (*n* = 2765; only SNPs with *p* < 1.0e‐5 were included). The original peripheral blood mQTLs data were generated in two cohorts: Brisbane Systems Genetics Study (BSGS) (*n* = 614) and Lothian Birth Cohorts (LBC) (*n* = 1366).^22^ The mQTLs summary data available here were a meta‐analysis of the BSGS and LBC data.^23^ Only the DNAm probes with at least a cis‐mQTL at *p* < 5.0e‐8 and only SNPs within 2 Mb distance from each probe were available.

### 2.3 Statistical analysis

#### 2.3.1 MR analysis

Our MR analysis was conducted following the guidelines outlined in the Strengthening the Reporting of Observational Studies in Epidemiology Using Mendelian Randomization (STROBE‐MR) checklist (Table S1).^24^, ^25^

For the aim of investigating the causal effect between GM and PC, we chose SNP to be used as IVs based on the following three major assumptions:

(1) The SNP must be related to the exposure factor to be studied (*p* < 1.0e−5). The 1000 Genomes project European samples data were used as a reference panel to calculate the linkage disequilibrium (LD) between SNPs. The LD was considered significant at an *r*^2 threshold of less than 0.01 within a 5000 kb clumping window. Additionally, the *F*‐statistic was used (*F*>10).

(2) The SNP cannot be associated with any confounders.

(3) The IVs exert a direct effect on the outcome via the exposure, independently of other variables. Confounded SNPs were removed by querying the literature and relevant databases (http://www.phenoscanner.medschl.cam.ac.uk/).

To evaluate the causal effect of GM on the risk of PC, we employed several MR methods, including IVW, MR‐Egger and weighted median (WM). IVW approach was chosen as the primary method for MR analysis because of its higher statistical efficacy, while the other two methods were used as complementary approaches. *p* < 0.05 was considered statistically significant as evidence of a potential causal effect. Heterogeneity was assessed by Cochrane's Q test and I^2, with *p* < 0.05 considered heterogeneous. Additionally, MR‐Egger regression was applied to examine the presence of horizontal pleiotropy in MR analysis. If *p* > 0.05, horizontal pleiotropy was considered not to be present. We analysed the pleiotropy using MR‐PRESSO and removed possible outliers to ensure the accuracy of the results (based on IVW results). We also conducted the Steiger test to assess the potential impact of reverse causation. Furthermore, sensitivity analysis was conducted by iteratively removing each SNP to implement the leave‐one‐out method, aiming to verify the reliability and stability of the estimated causal effects.

#### 2.3.2 SMR and colocalization analysis

The Multi‐SNP‐based SMR test was used to investigate the association between an exposure and a trait due to a shared variant at a locus and whether the effect of SNPs on the phenotype is mediated by gene expression.^11^ The integration of GWAS data with other molecular QTLs data by SMR analysis improved the detection of causal genes. Then we located the top SNPs in the cis‐region around the associated cis‐eQTLs and cis‐mQTLs, respectively. Additionally, we integrated the GM cis‐eQTLs and GM cis‐mQTLs. All the SNPs in a region passed a P threshold (*p* < 1.0e‐5) and used the false discovery rate (FDR) to adjust for multiple testing (FDR<0.05). The heterogeneity in dependent instruments (HEIDI) test was done to explore the existence of linkage in the observed association. Then those probes with little evidence of heterogeneity (P_HEIDI>0.05) were retained. We adopted the default settings in SMR [eQTL<1.0e‐5, mQTL<1.0e‐5, minor allele frequency (MAF)>0.01], excluding SNPs in very strong LD (*r*^2>0.9) with the top associated cis‐eQTLs or cis‐mQTLs.

Colocalization analysis was conducted between the GM cis‐eQTLs and PC cis‐eQTLs, and the GM cis‐mQTLs and PC cis‐mQTLs, to investigate potential gene interactions. It is the method to assess the presence of shared causal variants in the region for two traits. PPH4>0.5 is the threshold for the shared genetic effect between the two traits.

The regulatory signature enrichment of DNA methylation (DNAm) sites was assessed using eFORGE (http://eforge.cs.ucl.ac.uk/).

We performed MR and colocalization analysis mainly using the ‘TwoSample MR’ and ‘coloc’ packages, respectively, in R software (version 4.3.0). SMR analysis was conducted by SMR software (version 1.3.1) downloaded from the website (https://cnsgenomics.com/software/smr/).

## 3 RESULTS

### 3.1 The causal relationship between GM and PC

**FIGURE 2:**
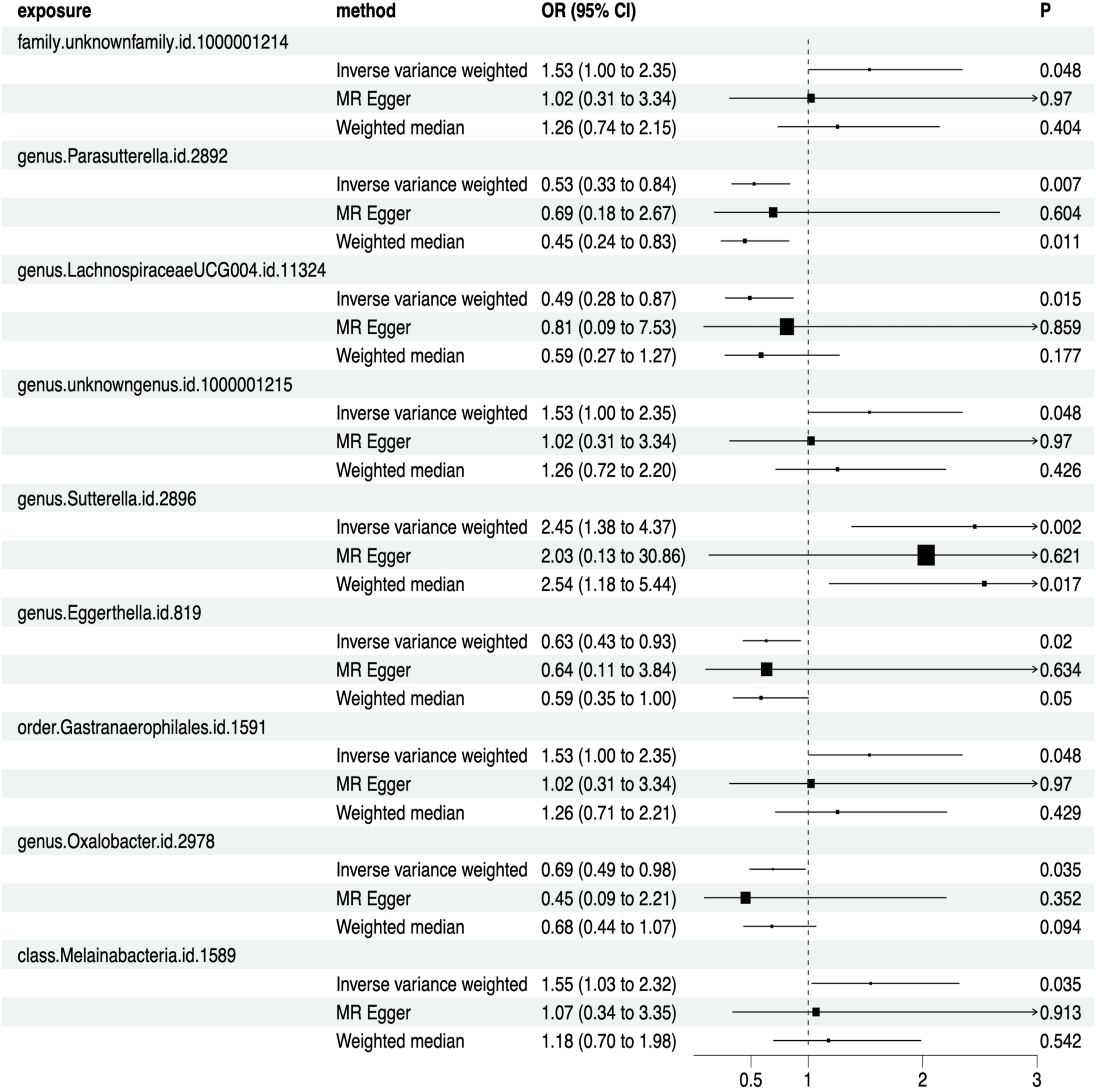
Causality of gut microbiota on pancreatic cancer.

**FIGURE 3:**
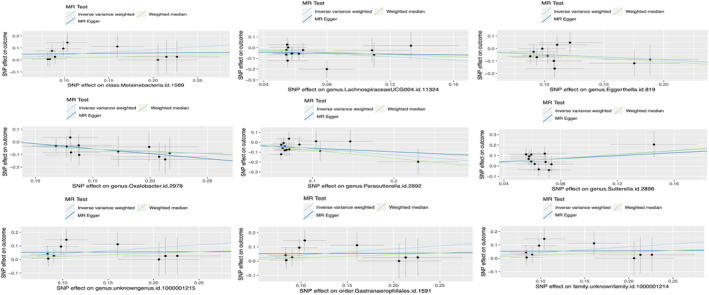
Mendelian randomization scatter plots. SNP, Single nucleotide polymorphism.

A total of 211 types of GM containing 14,581 SNPs (*p* < 1.0e‐5) were included. Based on the principles of IVs selection and local clumping (LD, *r*^2 < 0.01, window = 5000 kb), 2769 SNPs were identified as IVs. Finally, we detected nine types of GM (98 SNPs in total) that were causally associated with PC, including four protective and five risk‐associated GM (Figure 2). The effect of each associated SNP on the outcome was shown in Figure 3. Details can be seen in Table S2, and the F‐statistic for IVs was between 19.07 and 31.12 (Table S3), all over 10. Cochrane's Q test and I^2 for IVW and MR Egger showed no significant heterogeneity for these IVs (Cochrane's Q > 0.05, I^2 < 0.05; Table S4 and Table S5), and MR‐Egger regression intercept analysis found no horizontal pleiotropy (Table S6). The Steiger test showed that there was no reverse causal impact (Table S7). As for Sensitivity analysis in these causal effects, we detected no potential outliers in the leave‐one‐out plots (Figure S1) and further MR‐PRESSO analysis did not reveal any significant outliers either (Table S8).

### 3.2 SMR analysis and colocalization

In Multi‐SNP‐based SMR analysis, we identified 29 DNAm probes across three chromosomes by integrating GM GWAS data and blood cis‐mQTLs (FDR <0.05, P_HEIDI>0.05; Table S9) and detected 42 potential GM‐related genes in peripheral blood (FDR <0.05, P_HEIDI>0.05; Table S10). Additionally, SMR analysis of two molecular traits showed that there was only one putative GM‐effect gene: SVBP, also known as CCDC23, which was mediated by blood methylation regulation on gene expression (FDR <0.05, P_HEIDI >0.05; Table S11). These CpG sites were significantly enriched in the enhancer of transcription start sites (TSSs) of peripheral blood: Primary haematopoietic stem cells Granulocyte colony‐stimulating factor (G‐CSF) mobilized female (FDR = 0.013; Table S12). To further detect the interactions between GM and PC, we colocalized GM cis‐eQTLs with PC cis‐eQTLs and GM cis‐mQTLs with PC cis‐mQTLs, respectively. As a result, we detected that MCM6 and RPS26 were possible GM‐effect genes on PC based on the colocalization between GM cis‐eQTLs and PC cis‐eQTLs (Table S13). Otherwise, there was no overlapped genetic variant between GM cis‐mQTLs and PC cis‐mQTLs.

## 4 DISCUSSION

Our study employed the MR methods to investigate a causal relationship between GM and PC, aligning with the results of previous studies.^12^, ^13^, ^14^, ^15^, ^16^ They revealed various associations between different types of GM and PC due to the differences in the data sources used. Besides validating the causal relationship between GM and PC, we utilized SMR methods to integrate GM GWAS with blood cis‐eQTLs data and blood cis‐mQTLs data, respectively, aiming to identify genes in the blood tissue affected by GM. Additionally, we delved into the interacting genes between GM and PC through colocalization analysis.

The shared genes between GM and PC suggest that the microbiota may serve as a novel pathogenic factor. In murine models, GM originating from the intestine can contribute to carcinogenesis in the pancreatic duct.^26^ Additionally, a study of newly diagnosed, untreated patients with PDAC and matched controls indicated that specific faecal microbiota‐based screening for the early detection of PDAC, other than oral and tissue microbiota, is feasible.^27^ This suggested a role for the GM in the aetiology of PC. Microbiota can produce bacteria‐derived extracellular vesicles (EVs), which allow interaction with human cells.^28^ In a case–control study, compositional differences in microbiota, based on bacteria‐derived EVs extracted from blood serum, were identified as novel biomarkers.^29^ However, the study was limited by its small sample size, including only 38 patients with PC and 52 controls. The underlying mechanisms that were examined include the variability in microbiota composition,^30^, ^31^, ^32^ microbiota translocation,^26^, ^33^ microbiota‐immunity axis,^34^, ^35^ and the microbiota metabolites or metabolic pathways.^6^, ^36^, ^37^, ^38^ Despite these insights, the microbe‐host interactions as yet largely uncharacterized in PC.

Currently, an increasing number of biomarkers in blood tissue serve as monitoring, diagnosis, and prediction indices. Therefore, in this study, we conducted SMR analysis of two molecular traits using GM cis‐eQTLs data and GM cis‐mQTLs data. The DNAm probes were found in the enhancer region upstream of SVBP (CCDC23) and this DNAm was shown to negatively regulate its expression (b_SMR <0). Conversely, the expression level of SVBP (CCDC23) was positively associated with GM (b_SMR = 0.35). SVBP (CCDC23) enables microtubule‐binding activity. It was demonstrated that SVBP (CCDC23) formed a complex with Vasohibins (VASH1), leading to the dispersion in the cytosol and extracellular release of VASH1, which was identified as a regulator of angiogenesis and cancer cell functions,^39^ and premature stop codon SVBP variant impaired VASH1 secretion and solubility.^40^ The role of VASH/SVBP as the tubulin carboxypeptidase (TCP) implied that microtubule modifications might play an essential role in the epithelial‐to‐mesenchymal transition (EMT) process.^41^ Reviewed previous studies, it seems that VASH/SVBP as a complex played a role in carcinogenesis, rather than single gene SVBP.

We used the GM cis‐QTL data to colocalize the shared genes with PC, revealing MCM6 and RPS26 as interaction genes between GM and PC (PPH4>0.5). Peng et al. found a positive correlation between MCM6 and the proliferation marker Ki‐67, indicating a potential role in cellular growth.^43^ Elevated levels of MCM6 were notably linked to PC progression, aggressive PC cell behaviours, poorer disease‐free survival and poorer overall survival suggesting its significance as a prognostic marker, although it did not independently predict adverse outcomes in PC.^42^, ^43^ This study found that elevated expression of the MCM6 gene had adverse effects on PC (b_SMR = 0.18), aligning with findings from other studies.^42^ RPS26 encodes a ribosomal protein that is a component of the 40S subunit, and this protein, Rps26, is preferentially oxidized.^44^ Additionally, Rps26 directly binds to mRNA on the platform of the small (40S) subunit; this interaction assists in establishing the mRNA sequence preference during translation initiation, a process that is captured in the 5′ untranslated region of eukaryotic mRNA molecules.^44^ This study also found that elevated expression of the RPS26 gene is associated with adverse effects on PC (b_SMR = 0.12). Despite implying potential carcinogenic mechanisms, the association between RPS26 and PC is not well‐established and requires further investigation.

Since employing experimental methods to identify associations between GM and diseases can be costly and inefficient, computational models are often applied.^45^ These models effectively predict various associations between GM and non‐communicable diseases.^45^, ^46^, ^47^, ^48^ The use of computational models can offer a cost‐effective and efficient means to identify potential associations. It is feasible that the methods used in our study can provide support for these models at the genetic level.

This paper has some limitations. Firstly, we employed summary statistics instead of individual‐level data, which limited our ability to investigate non‐linear relationships. We were also not able to correct for sample overlap. Secondly, the predominant European ancestry of the GWAS participants may constrain the generalizability of our findings across different ethnic groups. Thirdly, we only focused on the cis‐regions in the analysis, despite the possibility that trans‐eQTLs SNPs may have a widespread impact on regulatory mechanisms. Fourthly, we utilized a Bayesian colocalization approach, predicated on the hypothesis that a single genetic variant influences two traits, given that the potential for multiple causal variants has yet to be extensively explored.^49^ Lastly, although our study, which integrated omics data, proposes the putative causal mechanisms, further experiments are still needed to validate our findings.

To conclude, we found a causal relationship between GM and PC. Furthermore, we explored the genes underlying the interactions between GM and PC. This study propels foundational research into the mechanism of GM on PC, pinpointing prospective novel diagnosis and therapeutic targets for clinical application.

## AUTHOR CONTRIBUTIONS

**Xin Li:** Conceptualization (lead); formal analysis (lead); methodology (lead); writing – original draft (lead); writing – review and editing (lead). **Zhihai Liang:** Funding acquisition (lead); project administration (lead); supervision (lead).

## FUNDING INFORMATION

This study was supported by the National Natural Science Foundation of China Projects (No.82360135).

## CONFLICT OF INTEREST STATEMENT

The authors confirmed that there are no conflicts of interest.

## Supporting information


Figure S1.

Table S1.

## Data Availability

The standard tools used in this study (including SMR) are available at https://yanglab.westlake.edu.cn/software/smr. GWAS summary statistics for GM and PC were downloaded from https://mibiogen.gcc.rug.nl/ and https://www.ebi.ac.uk/gwas/studies/GCST90041814, respectively. Blood eQTLs and mQTLs were obtained from https://yanglab.westlake.edu.cn/software/smr/#eQTLsummarydata and https://yanglab.westlake.edu.cn/software/smr/#mQTLs, respectively. PC cis‐mQTLs and cis‐eQTLs data can be found at http://gonglab.hzau.edu.cn/Pancan‐meQTL/cis and http://gonglab.hzau.edu.cn/PancanQTL/ respectively.
