# Supplementary figures and images for: Causal effect of gut microbiota on pancreatic cancer: A Mendelian randomization and colocalization study
Source: J Cell Mol Med. 2024 Mar 25;28(8):e18255. doi: 10.1111/jcmm.18255 (PMC10962122; doi:10.1111/jcmm.18255)

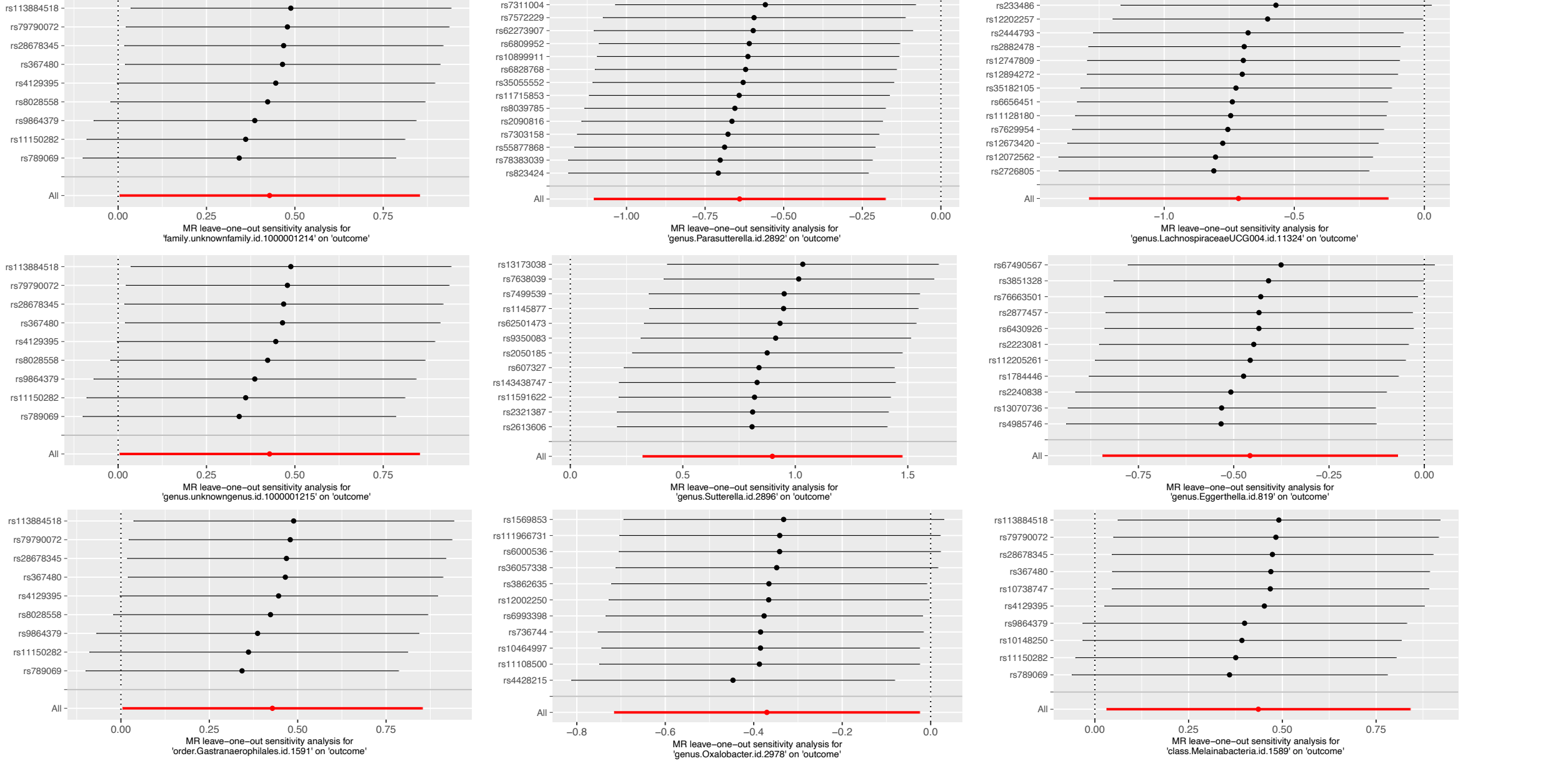

Figure S1. Leave-one-out plots.

Supplement: Supplementary file 1 — Figure S1. [file JCMM-28-e18255-s002.pdf]
